# The cannabinoid receptor CB1 affects the proliferation and apoptosis of adenomyotic human uterine smooth muscle cells of the junctional zone: a mechanism study

**DOI:** 10.1186/s12958-020-00690-0

**Published:** 2021-02-02

**Authors:** Sha Wang, Bohan Li, Xue Shen, Hua Duan, Zhengchen Guo, Xiao Li, Fuqing Sun

**Affiliations:** Department of Minimally Invasive Gynecologic Center, Beijing Obstetrics and Gynecology Hospital, Capital Medical University, 100006 Beijing, China

**Keywords:** Adenomyosis, Junctional zone, Cannabinoid receptor, CB1, Proliferation, Apoptosis

## Abstract

**Background:**

The denomyotic junctional zone (JZ) plays an important role in the pathogenesis of adenomyosis. Proliferating cell nuclear antigen (PCNA) is an important nuclear marker of cell proliferation. This study aimed to evaluate the effects of the cannabinoid receptor CB1 on proliferation and apoptosis in the JZ in women with and without adenomyosis.

**Methods:**

JZ smooth muscle cells (JZSMCs) of the adenomyosis and control groups were collected and cultivated. Immunohistochemistry and immunoblotting were used for protein localization and expression detection of CB1 and PCNA. Additionally, qRT-PCR was used to quantitatively analyse the mRNA expression of the two. AM251 and ACEA were used to regulate the function of CB1 receptors, and CCK-8 assay and flow cytometry assay were used to verify the proliferation and apoptosis of JZSMCs after regulation.

**Results:**

We demonstrated that in normal JZSMCs CB1 and PCNA messenger RNA (mRNA) and protein expression was significantly higher in the proliferative phase of the menstrual cycle than in the secretory phase. CB1 and PCNA expression in JZSMCs from women with ADS was significantly higher than that in control women and did not significantly differ across the menstrual cycle. CB1 receptor antagonist AM251 inhibited the proliferation of adenomyotic JZSMCs in a dose-dependent manner. The CB1 receptor agonist ACEA significantly promoted the proliferation of adenomyotic JZSMCs. The apoptosis rate of adenomyotic JZSMCs treated with AM251 was significantly higher than that of JZSMCs from the untreated control group. The apoptosis rate was significantly decreased in the ACEA group compared with that in the untreated control group. Furthermore, AM251 suppressed the phosphorylation of AKT and Erk1/2 in adenomyotic JZSMCs. The CB1 agonist ACEA significantly promoted the phosphorylation of AKT and Erk1/2.

**Conclusions:**

Our results indicated that the levels of CB1 and PCNA were increased in patients with adenomyosis and that cyclic changes were lost. CB1 may affect uterine JZ proliferation and apoptosis in adenomyosis by enhancing AKT and MAPK/Erk signalling.

## Introduction

Adenomyosis (ADS) is a common oestrogen-dependent uterine disorder distinguished by non-malignant invasion of the bioactive endometrium into the myometrial wall, and it can result in abnormal uterine bleeding, dysmenorrhoea, and subfertility [1, 2]. Junctional zone (JZ) dysfunction has been suggested as the causative factor in the development of ADS [3]. On T2-weighted magnetic resonance imaging (MRI) of the uterus, the JZ myometrium can be clearly distinguished from the endometrium and outer myometrium, and diffuse or focal thickening of this zone to a thickness greater than 12 mm has become the most widely accepted criterion for establishing the presence of ADS [4]. Our previous studies also found that in ADS the JZ showed cellular and nuclear hypertrophy, abnormal nuclear and mitochondrial morphology, abundant myelin bodies and intermediate filament aggregates, extensive endoplasmic reticulum, and lengthening of sarcolemma plaques [5]. However, the specific mechanism of the abnormal proliferation of JZ smooth muscle cells (JZSMCs) remains unknown.

Endocannabinoids were first discovered in the early 1990s [6]. The endocannabinoid system (ECS) comprises endogenously produced bioactive lipids, their molecular targets (two well-characterized G-protein–coupled cannabinoid receptors, CB1 and CB2), synthesis and degradation enzymes, and protein transporters [7, 8]. In recent years, the ECS has been reported to be involved in processes relevant to endometriosis, including cell migration, cell proliferation, apoptosis, and inflammation, and to interact with sex steroid hormones [9, 10]. However, such data on ADS are sparse. A large number of studies have confirmed that CB1 can affect the proliferation, migration and apoptosis of many types of cells by regulating the AKT and MAPK/Erk pathways (via phosphorylation activation) [11, 12]. In our previous study, we confirmed that expression of the cannabinoid receptors CB1 and CB2 was significantly higher in the ADS myometrium than in the normal myometrium, and CB1 expression in the JZ was positively correlated with the severity of dysmenorrhoea in patients with ADS [13]. However, whether the abnormally increased expression of CB1 is related to thickening of the JZ has not been reported. In addition, whether the increased activation of CB1 causes abnormalities in proliferation and apoptosis signalling is not entirely understood.

Based on these findings, it is reasonable to hypothesize that CB1 regulates proliferation in the JZ and participates in the pathogenesis of ADS. This study investigated the expression of CB1 and proliferating cell nuclear antigen (PCNA) in cultured JZSMCs from women with and without ADS. Furthermore, we observed the effect of CB1 on the proliferation and apoptosis of JZSMCs and related signalling molecules through pharmacological intervention of CB1 receptors.

## Methods

### Patients and specimens

The study was approved by the local research and ethics committee of the Beijing Obstetrics and Gynecology Hospital (No. 2016-KY-012). All patients included in this study signed an informed consent form. From March 2018 to July 2019, uterine samples were acquired at Beijing Obstetrics and Gynecology Hospital from two groups or patients. The control group consisted of 17 premenopausal women who underwent hysterectomy due to cervical intraepithelial neoplasm III (CIN III) at Beijing Obstetrics and Gynecology Hospital. Clinical examination of the control subjects confirmed regular menstruation with no evidence of ADS. The study group consisted of 21 premenopausal women who reported dysmenorrhoea or other symptoms of adenomyosis, and their histopathologic examination had been confirmed as ADS. Samples of the JZ were obtained as soon as the uterus was removed during surgery. Based on the reported thickness of the JZ in normal reproductive women [4], we took samples from 2 mm beneath the endometrium of the JZ.

All patients had normal menstrual cycles; no evidence of endometriosis, endometrial pathology or malignancy; and no history of intrauterine device placement or hormone therapy within three months before surgery. The ADS group consisted of 11 women in the proliferative phase and 10 women in the secretory phase, and the control group consisted of 10 women in the proliferative phase and 7 women in the secretory phase. The mean (standard deviation [SD]) ages of women in the case and control groups were 44.5 (SD = 4.3, range = 38–49) and 43.2 (SD = 3.6, range = 39–47) years, respectively. No significant difference in either age (*P* = 0.25) or menstrual cycle (*P* > 0.99) existed between the two groups. Cycle phase assignment was confirmed by endometrial histological assessment. The details are shown in Table 1.

**Table 1:** Overview of the demographics and other characteristics of the recruited patients

Parameter	Adenomyosis N = 21	Control N = 17	*P*
Age, mean (SD)	44.5 (4.3)	43.2 (3.6)	*P* = 0.25
Menstrual cycle (Proliferative phase) n (%)	11 (52.4%)	10 (58.8%)	*P* > 0.99
Uterine fibroids n (%)	8 (38.1%)	4 (23.5%)	*P* > 0.53
Gravidity, median (range)	2 (0, 5)	2 (1, 5)	*P* = 0.75
Parity, median (range)	1 (0, 2)	1 (0, 2)	*P* = 0.59
PBAS, mean (SD)	158.7 (67.6)	112.9 (25.9)	*P* = 0.12
Chronic medication n	0	0	*P* > 0.99
Previous uterine surgery n	0	1	*P* = 0.46

### Cell culture and drug treatment

Fresh junctional myometrium was obtained from the uterine samples within 10 min of surgical removal and opened in the sagittal plane. The tissue was collected in ice-cold saline, washed, and processed for cell dispersion. The endometrium was removed by gentle scratching at the endometrial-myometrial interface (EMI) with a surgical blade. Human uterine JZSMCs were cultured and identified as previously described, and cells at passages 2 to 6 were used [14–16]. To prevent contact inhibition or promotion, cells at no more than 75% confluence were starved of endogenous steroids for 24 hours in phenol red-free Dulbecco’s modified Eagle medium (Gibco, Grand Island, New York) supplemented with 2% charcoal-stripped foetal bovine serum (Biosera, Kansas City, Missouri). Then, cells in ‘‘starvation medium’’ were exposed to the following compounds: the CB1 receptor antagonist AM251 (Sigma-Aldrich, St. Louis, Missouri), and the CB1 receptor agonist ACEA (Sigma-Aldrich).

### Immunohistochemistry

Immunohistochemistry was performed as described previously [17]. Sections (4 µm) were prepared from each sample, dewaxed in xylene, rehydrated with a graded ethanol series and rinsed in distilled water. For antigen retrieval, the sections were boiled in citric saline (10 mmol/L, pH 6.0) for half an hour. Then, the samples were treated with a 3% hydrogen peroxide solution for 25 min to block endogenous peroxidase activity. After the samples were blocked with 3% bovine serum albumin (BSA, Servicebio, Wuhan, China) for 30 min at room temperature, they were incubated with either rabbit anti-CB1 antibody (Cell Signaling Technology, diluted 1:300) or mouse anti-PCNA antibody (Cell Signaling Technology, diluted 1:100) at 4 °C overnight. For the negative controls, phosphate-buffered saline (PBS) was used instead. Next, the sections were rinsed in PBS 3 times and incubated with horseradish peroxidase-labelled goat anti-rabbit antibody (Servicebio; diluted 1:200) for 50 min at room temperature. After being washed with PBS and incubated with 3,3’-diaminobenzidine tetrahydrochloride dihydrate (Servicebio), the sections were counterstained with haematoxylin for 3 min. Finally, all slides were mounted on glass slides with Permount (Servicebio), examined with a Leica DM4000B microscope (Leica, Wetzlar, Germany) and imaged with Leica Application Suite (LAS, version 4.9.0, Leica). The immunohistochemical staining results were assessed with Image-Pro Plus 6.0 software (Media Cybernetics, Rockville, Maryland) as reported previously [18]; the evaluators were blinded to the patients’ information. A series of 10 images of each section for each targeted protein were randomly extracted to obtain an average value for statistical comparison. Colour intensity was used to define the staining intensity, and a colour mask was generated. Then, the mask was equally applied to all images, and measurements were acquired. The mean optical density (MOD), defined as the ratio of the integrated optical density (IOD) to the total stained area, was recorded and considered to be equivalent to the immunoreactivity level of the target substance in the endometrium.

### Immunoblotting

JZSMCs were harvested, counted, and lysed in ice-cold Frackelton buffer [10 mM Tris-HCl (pH 7.05), 50 mM NaCl, 30 mM sodium pyrophosphate, 50 mM NaF, 1% Triton X-100, 1 Na_3_VO_4_, one protease inhibitor cocktail tablet (Roche), and 1 mM phenylmethylsulfonyl fluoride]. Insoluble material was removed by centrifugation of the samples at 20,000 × *g* for 20 min at 4 °C. Cytosolic and nuclear cellular lysates were prepared using NE-PER Nuclear and Cytoplasmic Extraction Reagents from a kit (Pierce Chemical Co., Colfax, CA, USA) according to the manufacturer’s protocol. The protein concentrations in the total-cell lysates and cytosolic or nuclear extracts were determined using the bicinchoninic acid protein assay (Thermo Scientific, IL, USA). Twenty micrograms of each normalized sample was immunoblotted as previously described ^9^ and probed with the following primary antibodies: anti-CB1, anti-PCNA (BD Biosciences, CA, USA), anti-AKT, anti-p-AKT (Santa Cruz Biotechnology, Santa Cruz, CA, USA), anti-Erk1/2, anti-p-Erk1/2 (Cell Signalling Technology, Danvers, MA, USA), and anti-β-actin (Sigma-Aldrich). The blots were then incubated with HRP-conjugated secondary antibody (Pierce Chemical Company, Rockford, IL, USA) for 1 hour at room temperature. Detection of the reactive antigens was performed with an ECL kit (Amersham Life Sciences, Inc., Arlington Heights, IL, USA), followed by exposure of the membrane to X-ray film. The resulting images were analysed with a ChemiImager 4000 system (Alpha Innotech Corporation, San Leandro, CA, USA) to determine the integrated density value (IDV) for each protein band normalized to the IDV for β-actin. All data are presented as the mean of three independent experiments.

#### Quantitative real‐time polymerase chain reaction (qRT-PCR)

Total RNA was extracted from the JZSMCs with RNAiso Plus (Takara Bio, Inc., Shiga, Japan) and quantified with a NanoDrop 2000/2000c spectrophotometer (Thermo Fisher Scientific Inc., Massachusetts, USA). A PrimeScriptTM RT Reagent Kit (RR047A, Takara) was used to synthesize cDNA from 1 µg of total RNA per sample. The primers used in this study were designed by Sangon Biotech Co., Ltd. (Shanghai, China), and their sequences are presented in Table 2. PCR was performed on an ABI 7500 real-time polymerase chain reaction system (Applied Biosystems, Grand Island, New York) with the protocol for SYBR Premix Ex TaqTM II (RR820A, Takara). The reaction protocol consisted of 95 °C for 30 seconds for initial denaturation followed by 40 cycles of 5 seconds at 95 °C and 34 seconds at 60 °C. The 2^-△CT^ method was used to analyse relative gene expression as reported^19^ in which ΔCT = CT (target gene) – CT (internal control).

**Table 2:** Specific primers used for qRT-PCR analysis

Gene		Sequence
**CB1**	Forward	5′-CCTAGATGGCCTTGCAGATACC-3′
	Reverse	5’-GAATGTCATTTGAGCCCACGTA-3′
**PCNA**	Forward	5′-TTCGA ACCTACCGCTGC-3′
	Reverse	5′-TCTCCTGGTTTGGTGCTTC-3′
**β-actin**	Forward	5′-TGCCGACAGGATGCAGAAG-3′
	Reverse	5′-CTCAGGAGGAGCAATGATCTTGA-3′

### CCK-8 assays

CCK-8 (Dojindo Technology Co., Ltd.) assays were used to assess cell proliferation and cell viability. JZSMCs were cultured in 96-well plates at a density of 4,000 cells/well and incubated at 37 °C in 5% CO_2_. CCK-8 solution (10 µl) was added to each well at different times and incubated for 4 hours, after which the plate was shaken for 1 min. Finally, the absorbance at 450 nm was measured with a microplate reader.

### Flow cytometry assay

After serum starvation followed by inhibitor or agonist treatment, cells seeded in 6-cm dishes were harvested and fixed in ice-cold 70% ethanol at 4 °C overnight. On the following day, fixed cells were incubated in 500 mL of propidium iodide/RNase staining buffer (BD Biosciences, San Jose, California) for 15 min in the dark at room temperature. The stained cells were then analysed using a Gallios flow cytometer (Beckman Coulter, Brea, California). Cell debris and fixation artefacts were gated out, and the percentage of cells in each phase of the cell cycle was quantified using ModFit 4.0 software. At least 10,000 cells in each sample were analysed to obtain a measurable signal.

### Statistical analysis

The statistical analysis was performed using Statistical Product and Service Solutions (SPSS) 22.0. After the normality of the data distribution was verified by the Kolmogorov-Smirnov test, the mean ± SD from three independent replicates of each experiment was calculated. The independent t-test was used to compare two groups. One-way analysis of variance (ANOVA) was applied to assess the statistical significance of differences between more than two groups. Before ANOVA or the independent t-test, homogeneity of variance between groups was analysed using the F test. Differences for which P < 0.05 were considered statistically significant.

## Results

### CB1 and PCNA protein expression levels in the adenomyotic JZ

**Fig. 1:**
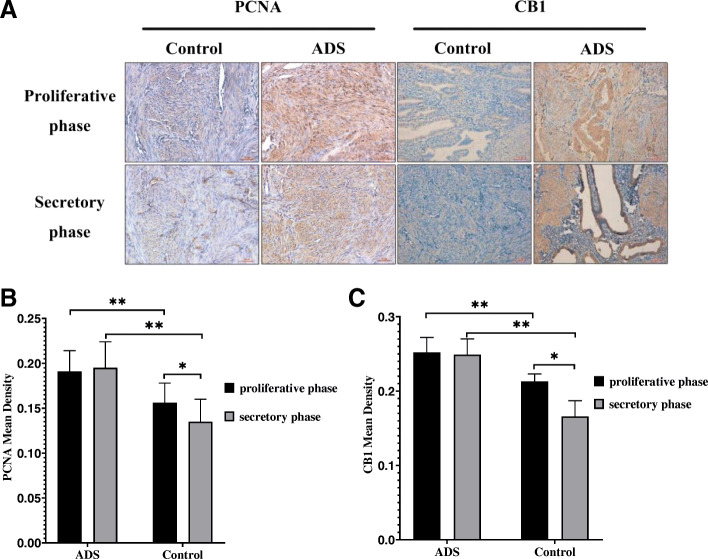
Immunohistochemical staining for CB1 and PCNA. **a** CB1 and PCNA expression in women in the adenomyosis group and control group in the proliferative and secretory phases. **b** Quantitative analysis of the mean optical density (MOD) of CB1 between the proliferative phase and secretory phase in the adenomyotic JZ and control JZ. **c** Quantitative analysis of the mean optical density (MOD) of CB1 between the proliferative phase and secretory phase in the adenomyotic JZ and control JZ. All micrographs are magnified 200×. Scale bars represent 5 µm. The error bars on all histograms indicate the standard deviation. *, *P* < 0.05; **, *P* < 0.01

PCNA is an important nuclear marker of cell proliferation. In the ADS and control groups, PCNA and CB1 expression was observed in endometrial glandular epithelial cells, stromal cells, vascular endothelial cells, JZSMCs and ectopic endometrial cells (Fig. 1a). Brown PCNA- and CB1-positive granules were observed in the cytoplasm and cell nuclei. Immunohistochemical staining was preliminarily performed to investigate the expression of CB1 and PCNA in the JZ myometrium in the two groups. The MOD of CB1 and PCNA was significantly increased in the adenomyotic JZ compared with that in the normal JZ (for the proliferative phase, both *P* < 0.001; for the secretory phase, both *P* < 0.001). CB1 and PCNA immunoreactivity in the control JZ was higher in the proliferative phase of the menstrual cycle than in the secretory phase (*P* < 0.05). However, cyclic variation in CB1 and PCNA immunoreactivity was not observed in the adenomyotic JZ (*P* = 0.087 and *P* = 0.916, respectively) (Fig. 1b and c).

### CB1 and PCNA protein expression levels in adenomyotic JZSMCs

**Fig. 2:**
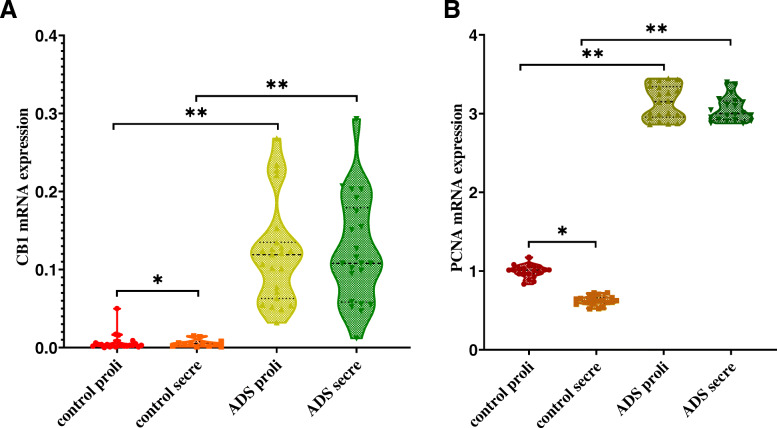
CB1 and PCNA protein expression in JZSMCs from normal and adenomyotic women across different phases of the menstrual cycle was determined by Western blotting. **a** Proteins were extracted from the JZSMCs of women in both groups in the secretory phase (Secre) and proliferative phase (Proli) and subjected to Western blot analysis. β-Actin was assayed in parallel as an internal control. **b** Quantitative analysis of average CB1 and PCNA protein levels in each group normalized to β-actin. Three independent experiments were conducted for each treatment. One representative image from each group of three experiments is shown. The error bars on all histograms indicate the standard deviation. *, *P* < 0.05; **, *P* < 0.01

The Western blotting results showed no significant differences in CB1 or PCNA protein expression in JZSMCs in the ADS group between the proliferative and secretory phases (*P* > 0.05). In the control group, the expression levels of CB1 and PCNA in the proliferative phase were significantly greater than those in the secretory phase (*P* < 0.05). The CB1 and PCNA levels in both the proliferative and secretory phases were higher in the JZSMCs of the ADS group than in those of the control group (for the proliferative phase, *P* < 0.05, *P* < 0.01, respectively; for the secretory phase, *P* < 0.05, *P* < 0.01, respectively) (Fig. 2a and b).

### CB1 and PCNA mRNA expression levels in adenomyotic JZSMCs

**Fig. 3:**
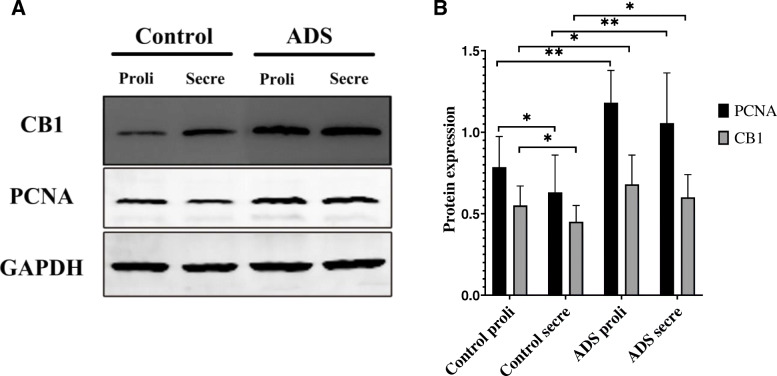
CB1 and PCNA mRNA expression in JZSMCs from control and adenomyotic women in the proliferative phase (Proli) and secretory phase (Secre). Three independent experiments were conducted for each treatment. One representative image from each group of three experiments is shown. The error bars on all histograms indicate the standard deviation. *, *P* < 0.05; **, *P* < 0.01

To further determine the CB1 and PCNA expression levels in the JZSMCs of women with ADS and control women, we observed the CB1 and PCNA messenger RNA (mRNA) expression levels using qRT-PCR and found them to be similar. In the control group, CB1 and PCNA mRNA expression was significantly higher in the proliferative phase than in the secretory phase (*P* < 0.05). In the ADS group, no significant difference in CB1 or PCNA mRNA expression between the secretory and proliferative phases was observed (*P* > 0.05). The comparison of CB1 and PCNA mRNA expression in JZSMCs showed significantly higher transcript levels in the ADS group than in the control group (*P* < 0.05) (Fig. 3a and b).

### Effect of pharmacological intervention of the CB1 receptor on the proliferation of JZSMCs in the adenomyotic uterus

**Fig. 4:**
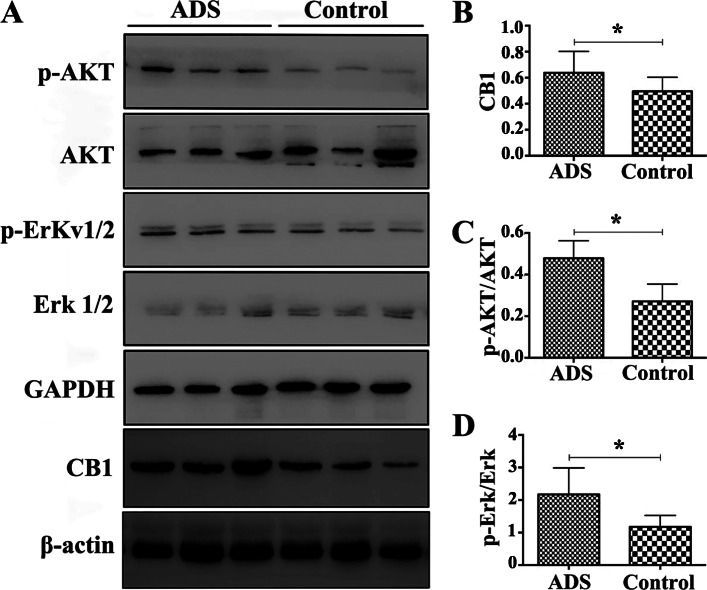
Expression of the CB1, AKT, p-AKT, Erk1/2 and p-Erk1/2 proteins in JZSMCs. **a** Characteristic blots for each protein. **b** Comparison of intercellular CB1 protein expression in each group. **c** Comparison of the intercellular p-AKT/AKT ratios. **d** Comparison of the intercellular p-Erk/Erk ratios. Three independent experiments were conducted for each treatment, and one representative image from each group of three experiments is shown. The error bars on all histograms indicate the standard deviation. *, *P* < 0.05

**Fig. 5:**
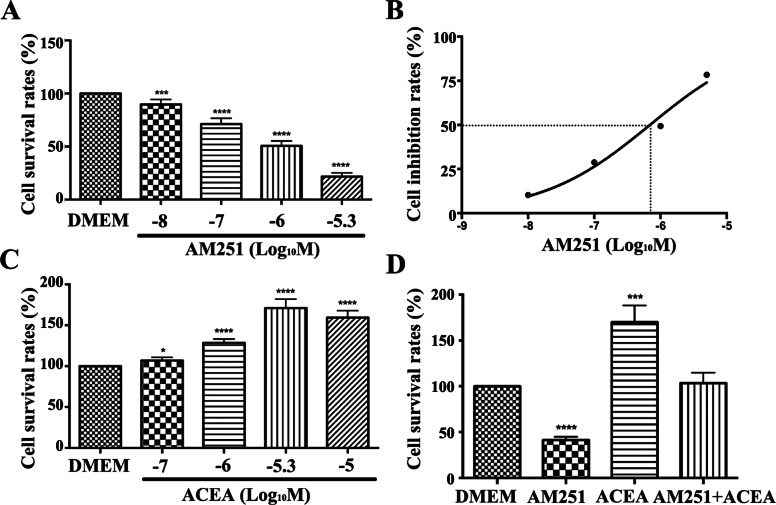
Effects of a CB1 receptor antagonist and agonist on the proliferation of JZSMCs. **a** JZSMCs were treated with the CB1 receptor antagonist AM251 at different concentrations, and the cell survival rates were analysed. **b** Inhibition effect of AM251 at different concentrations on adenomyotic JZSMCs. The X axis corresponds to the 50% inhibition rate (the logarithm (base ten) of the half maximal inhibitory concentration (IC50)). **c** The cell survival rates after incubation with the CB1 receptor agonist ACEA at different concentrations are shown. **d** Comparison of the cell survival rates after incubation with different drugs. Three independent experiments were conducted for each treatment. One representative image from each group of three experiments is shown (Log_10_ M is the logarithm of the molar concentration to 10, * *P* < 0.05; ** *P* < 0.01, ***, *P* < 0.001, ****, *P* < 0.0001)

To elucidate the role of CB1 in the proliferation of human uterine JZSMCs, we stimulated and inhibited the activity of CB1 in adenomyotic JZSMCs and then detected their capacity for cell proliferation. The CCK-8 assay showed that the CB1 receptor antagonist AM251 inhibited the proliferation of adenomyotic JZSMCs in a dose-dependent manner. The cell survival rate was significantly decreased by AM251 at all concentrations in the physiological range that were tested (10^− 8^ mol/L, 10^− 7^ mol/L, 10^− 6^ mol/L, and 5 × 10^− 6^ mol/L) (*P* < 0.05, Fig. 4a). The IC_50_ of AM251 in the JZSMCs was 6.931 × 10^− 7^ mol/L, as shown by regression analysis with GraphPad Prism 6.0 software; thus, the optimal concentration of AM251 used in the following experiment was 10^− 6^ mol/L (Fig. 5b).

**Fig. 6:**
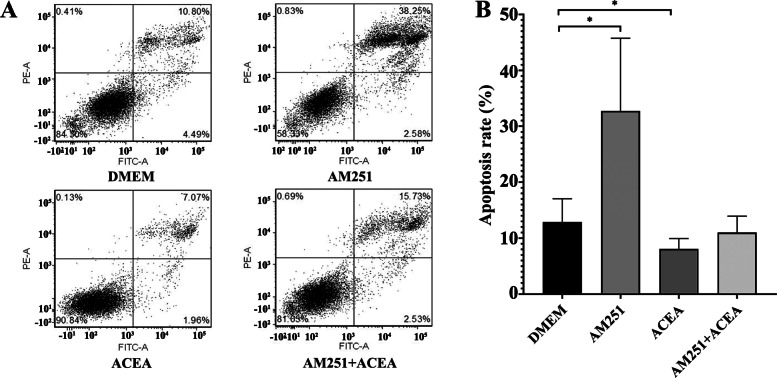
Effects of pharmacological intervention of CB1 on the apoptosis of JZSMCs in adenomyosis. **a** Flow cytometry data from cells treated with serum-free medium containing phenol red, 10^− 6^ mol/L AM251, 5 × 10^− 6^ mol/L ACEA, or 10^− 6^ mol/L AM251 for 30 min followed by 5 × 10^− 6^ mol/L ACEA. **b** Comparison of the apoptosis rates after treatment with different drugs (*, *P* < 0.05) ***、***

The CB1 receptor agonist ACEA promoted the proliferation of JZSMCs in the ADS group. After treatment with ACEA at different concentrations (10^− 7^ mol/L, 10^− 6^ mol/L and 5 × 10^− 6^ mol/L), the cell survival rate increased with increasing ACEA concentration; meanwhile, the cell survival rate at 10^− 5^ mol/L was significantly lower than that of the group treated with 5 × 10^− 6^ mol/L ACEA (*P* < 0.01, Figs. 3, 4, 5 and 6c). Therefore, the optimal concentration of ACEA used in the following experiment was 5 × 10^− 6^ mol/L (Fig. 4c).

To further clarify the role of the CB1 receptor, adenomyotic JZSMCs were divided into four groups for pharmacological interference with the CB1 receptor. The four groups were pretreated with serum-free medium containing phenol red (control group), 10^− 6^ mol/L AM251 (AM251 group), 5 × 10^− 6^ mol/L ACEA (ACEA group) or 10^− 6^ mol/L AM251 for 30 min followed by 5 × 10^− 6^ mol/L ACEA (combination group). The cell survival rate of the control group was set as 100%. The cell survival rate in the ACEA group was 169.9 ± 18.3%, which was significantly higher than that in the control group (*P* < 0.001). The cell survival rate in the AM251 group was 41.5 ± 3.5%, which was significantly lower than that in the control group (*P* < 0.001). No significant difference in cell survival rate was found between the combination group and the control group (*P* > 0.05) (Fig. 5d).

### Effect of pharmacological intervention of the CB1 receptor on JZSMC apoptosis in adenomyotic uteri

A flow cytometry assay showed that the apoptosis rate of JZSMCs after treatment with AM251 was significantly higher in the ADS group than in the control group (*P* < 0.001). The apoptosis rate in the ACEA group was significantly lower than that of the control group (*P* < 0.001). No significant difference in apoptosis rate was found between the combination group and the control group (*P* > 0.05) (Fig. 5a and b).

### Evidence for the involvement of CB1 in activation of the AKT/Erk pathway in adenomyotic JZSMCs

**Fig. 7:**
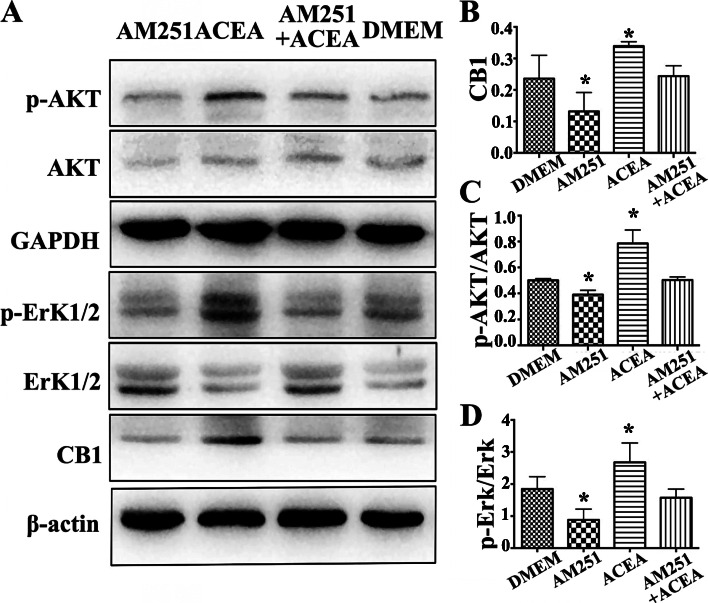
Effect of pharmacological intervention of CB1 on expression of the CB1, AKT, p-AKT, Erk1/2 and p-Erk1/2 proteins in adenomyotic JZSMCs. **a** Characteristic blots for each protein. **b** Comparison of intercellular CB1 protein expression in each group. **c** Comparison of the intercellular p-AKT/AKT ratios. **d** Comparison of the intercellular p-Erk/Erk ratios. Three independent experiments were conducted for each treatment, and one representative image from each group of three experiments is shown. The error bars on all histograms indicate the standard deviation. *, *P* < 0.05

The AKT signalling pathway plays a vital role in regulating cell progression and inhibiting pro-apoptotic protein expression. We aimed to investigate whether CB1 is involved in the activation of AKT in adenomyotic JZSMCs. Immunoblotting showed that the CB1 antagonist AM251 suppressed the phosphorylation of AKT and Erk1/2 in adenomyotic JZSMCs. The CB1 receptor agonist ACEA significantly promoted the phosphorylation of AKT and Erk1/2. The expression of CB1 and the phosphorylation of AKT and Erk1/2 did not significantly differ between the combination group and control group, which further suggested that CB1 is involved in activation of the AKT/Erk pathway (Fig. 7a-d).

## Discussion

Abnormal thickening of the JZ can influence sperm transport and embryo implantation and increase intrauterine pressure, resulting in infertility, dysmenorrhoea and menorrhagia. [19, 20]. Our previous electron microscopic research showed that adenomyotic JZSMCs exhibit hyperplasia, and analysis of the ultrastructure indicated the loss of cyclical changes [5]. Exploration of the specific manifestations and mechanism of proliferation dysfunction in JZSMCs is of great importance in uncovering the pathogenesis of ADS. PCNA is an important nuclear marker of cell proliferation [21]. In this study, we first detected PCNA expression in the JZ from specimens obtained from both ADS and non-ADS patients to reveal the proliferative characteristics of JZ. PCNA showed persistently high expression in the JZ, and cyclical changes were lost, which may be related to irregular thickening in ADS.

In our study, the proliferative capacity of JZSMCs in the ADS group was higher than that in the control group, which also explained the abnormal thickening of the JZ observed in ADS. Through molecular biology technology, we further demonstrated that CB1 expression in JZSMCs differed between the ADS and control groups; the protein and gene expression of CB1 was significantly higher in the ADS group than in the control group. The adenomyotic uterus has been previously reported to exhibit obvious myocyte hypertrophy, in which cytoplasmic aggregates were formed by abundant intermediate filaments, the edge of the nucleus was clear, the nucleolus and surrounding nuclear chromatin were obvious, and the rough endoplasmic reticulum and Golgi complex were more prominent than those in the absence of ADS, indicating that protein synthesis was more active; all of these characteristics were more pronounced in the JZ [22]. This may be the material basis for CB1 upregulation as well as the theoretical basis for the experimentally observed JZSMC hypertrophy in the ADS group. In 2004, Dennedy et al. [23] demonstrated the presence of the cannabinoid receptors CB1 and CB2 in human uterine smooth muscle for the first time. They studied myometrial biopsy specimens from caesarean section at term and found that both endogenous and exogenous cannabinoids could mediate the relaxation of human uterine smooth muscle in vitro during pregnancy by the CB1 receptor rather than the CB2 receptor. Uterine leiomyoma and ADS are oestrogen-dependent diseases, and a recent study showed no significant difference in CB1 expression between fibroid tissue and the normal myometrium, which seems inconsistent with our findings. However, the specimens used in that report were from patients aged 37 to 77 years, which was not appropriate for the study of uterine fibroids. For endometriosis, which exhibits pathological characteristics similar to those of ADS, studies have shown that activation of CB1 could promote the growth of lesions in endometriosis animal models and the migration of endometrial stromal cells [24–27]. A study also reported that the expression of endogenous cannabinoids and their related intermediates was significantly increased in ADS patients during the menstrual cycle [26], which is consistent with our finding that the selective CB1 receptor agonist ACEA promoted the proliferation of JZSMCs.

We observed the expression levels of AKT and MAPK/Erk pathway proteins in the ADS and control groups by Western blotting to further identify the cause of differences in the in vitro proliferative capacity of the two groups. The total AKT and total Erk1/2 expression in the ADS group was comparable to that in the control group, but the expression of p-AKT and p-Erk1/2 significantly differed. The analysis revealed that both the p-AKT/AKT and p-Erk/Erk ratios in the ADS group were significantly higher than those in the control group, suggesting abnormal activation of the AKT and MAPK/Erk pathways in the abnormally proliferating cells of the ADS group.

Studies have shown that activation of the AKT and MAPK/Erk pathways not only promotes the proliferation and migration of endometrial stromal cells in endometriosis [28, 29] but also participates in the progesterone resistance of endometriosis [30], suggesting that the AKT and MAPK/Erk pathways are potential targets for endometriosis treatment. The current study demonstrates that the AKT and MAPK/Erk pathways are also abnormally activated in aberrantly proliferating adenomyotic JZSMCs, suggesting that the AKT and MAPK/Erk pathways are therapeutic targets for ADS.

The CCK-8 assay results showed that the CB1 receptor antagonist AM251 dose-dependently inhibited the proliferation of JZSMCs in uterine ADS in vitro. Decreased cell survival was observed with increasing antagonist concentration. When JZSMCs were pretreated with AM251 before treatment with ACEA, the cell survival rate was comparable to that of the untreated group, suggesting that AM251 can completely antagonize the pharmacological activation of CB1 by ACEA. This finding is essentially consistent with the result reported by Eckardt et al., who showed that AM251 could also completely block the effect of arachidonoyl ethanolamine or anandamide (AEA, an endocannabinoid) in human skeletal muscle cells [31].

In the present study, the rate of apoptosis was analysed using flow cytometry. The results demonstrated that the selective CB1 receptor antagonist AM251 could promote the apoptosis of JZ smooth muscle cells in uterine ADS and completely block the inhibitory effect of its agonist, ACEA. Similar to the role of the CB1 receptor in cell proliferation, previous studies on the role of the CB1 receptor in apoptosis have suggested that it plays a dual role in apoptosis. Caunt et al. [32] found that ACEA promoted the survival of nerve cells through upregulating expression of the apoptosis suppressor protein Bcl-2. However, a contradictory result was reported by Gurbuz et al. [33], who showed that AM251 induced cell apoptosis in A375 human melanoma cells through downregulating expression of the apoptosis suppressor proteins Bcl-2 and survivin and upregulating the expression of Bax.

To further elucidate the specific mechanism of the CB1 receptor in the proliferation and apoptosis of JZSMCs, the selective CB1 receptor agonist ACEA and antagonist AM251 were used for pharmacological intervention of the CB1 receptor. ACEA activated the intracellular CB1 receptor and upregulated the expression of p-AKT and p-Erk1/2 while AM251 could completely antagonize the effect of ACEA, which suggested that the expression of CB1 and the p-AKT/AKT and p-Erk/Erk ratios were not significantly different from those of the untreated group after pretreatment with AM251 and the subsequent ACEA treatment. These results suggested that the CB1 receptor can act on JZSMCs by activating AKT and the MAPK/Erk signalling pathway and that the selective CB1 receptor antagonist AM251 has potential therapeutic applications for ADS.

As a preliminary study, this report has several possible limitations. First, this study is not based on a fairly large sample size of patients, especially when detecting the impact of CB1 on the function of JZ in different phases of the menstrual cycle in ADS. Second, we did not discover the relationship of CB1 with the clinical characteristics of adenomyosis (focal and diffused) and potential cytokines, immune cells, and other potential molecular pathways.

In conclusion, we confirmed that uterine JZSMCs in the ADS displayed significantly greater proliferative ability than those in the control group. The cannabinoid receptor CB1, the major receptor involved in endocannabinoid function, promoted the proliferation of JZSMCs in uterine ADS and inhibited apoptosis by activating the AKT and MAPK/Erk signalling pathways. Our study on the role of the CB1 in adenomyosis will be helpful for determining whether the endocannabinoid system could be a novel target for treating adenomyosis. However, further research is still needed to better clarify this system in ADS.

## Data Availability

All analysis results are displayed in the results. For specific experimental data, please contact the corresponding author.

## Abbreviations

ADS: Adenomyosis
JZ: Junctional zone
PCNA: Proliferating cell nuclear antigen
JZSMCs: JZ smooth muscle cells
mRNA: Messenger RNA
MRI: Magnetic resonance imaging
ECS: Endocannabinoid system
CIN III: Cervical intraepithelial neoplasm III
EMI: Endometrial-myometrial interface
qRT-PCR: Quantitative real-time polymerase chain reaction
